# State-of-the-Science Workshop Report: Issues and Approaches in Low-Dose–Response Extrapolation for Environmental Health Risk Assessment

**DOI:** 10.1289/ehp.11502

**Published:** 2008-09-19

**Authors:** Ronald H. White, Ila Cote, Lauren Zeise, Mary Fox, Francesca Dominici, Thomas A. Burke, Paul D. White, Dale B. Hattis, Jonathan M. Samet

**Affiliations:** 1 Johns Hopkins Bloomberg School of Public Health, Baltimore, Maryland, USA; 2 U.S. Environmental Protection Agency, Research Triangle Park, North Carolina, USA; 3 California Environmental Protection Agency, Office of Environmental Health Hazard Assessment, Sacramento, California, USA; 4 U.S. Environmental Protection Agency, Washington, DC, USA; 5 Clark University, Worcester, Massachusetts, USA

**Keywords:** dose–response function, environmental health, low-dose extrapolation, risk assessment, workshop report

## Abstract

Low-dose extrapolation model selection for evaluating the health effects of environmental pollutants is a key component of the risk assessment process. At a workshop held in Baltimore, Maryland, on 23–24 April 2007, sponsored by U.S. Environmental Protection Agency and Johns Hopkins Risk Sciences and Public Policy Institute, a multidisciplinary group of experts reviewed the state of the science regarding low-dose extrapolation modeling and its application in environmental health risk assessments. Participants identified discussion topics based on a literature review, which included examples for which human responses to ambient exposures have been extensively characterized for cancer and/or noncancer outcomes. Topics included the need for formalized approaches and criteria to assess the evidence for mode of action (MOA), the use of human versus animal data, the use of MOA information in biologically based models, and the implications of interindividual variability, background disease processes, and background exposures in threshold versus nonthreshold model choice. Participants recommended approaches that differ from current practice for extrapolating high-dose animal data to low-dose human exposures, including categorical approaches for integrating information on MOA, statistical approaches such as model averaging, and inference-based models that explicitly consider uncertainty and interindividual variability.

Over the past half-century, methodologic advances have provided an increasingly strong quantitative basis for estimating the human health risks associated with exposures to environmental contaminants. Estimation of the dose–response function is one of four critical elements of the now paradigmatic approach to health risk assessment developed in 1983 by the National Research Council (NRC 1983). Establishing dose–response functions frequently requires extrapolating limited amounts of data from high-concentration animal toxicologic studies to the relatively lower concentrations typically experienced by humans. Statistical methods, known as “low-dose extrapolation” models, have been developed for this purpose, and their merits and limitations have been debated since the earliest efforts in environmental contaminant risk assessment.

Recent advancements in statistical methods have allowed for more robust epidemiologic evaluation of very large populations exposed to environmental pollutants at ambient concentrations, thus providing information that informs low-dose extrapolation issues. In studied populations, thresholds have not generally been observed for cancer or, more notably, noncancer outcomes. This observation derives primarily from studies of radiation (NRC 1999, 2005), secondhand tobacco smoke [U.S. Department of Health and Human Services (U.S. DHHS) 2004], nitrogen and sulfur oxides [U.S. Environmental Protection Agency (EPA) 2008a, 2008b], particulate matter (U.S. EPA 2006b), ozone (U.S. EPA 2006a), and lead (U.S. EPA 2006c). These studies have spurred reconsideration of the cancer and noncancer paradigms used to extrapolate dose–response relationships for the relatively low doses of environmental toxicants typically encountered in the ambient environment.

The U.S. EPA and the Johns Hopkins Risk Sciences and Public Policy Institute (RSPPI) organized a workshop, titled “State-of-the-Science Workshop: Issues and Approaches in Low Dose–Response Extrapolation for Environmental Health Risk Assessment,” held 23–24 April 2007 in Baltimore, Maryland. Participants included 17 experts from diverse disciplines, including toxicology, biostatistics, human biology, epidemiology, and risk assessment (see Appendix 1). Workshop background materials, prepared by RSPPI, focused on a literature review on low-dose extrapolation, including extensively characterized examples of observed human responses at low ambient exposure levels. Workshop goals were to *a*) review the state of the science for high- to low-dose–response extrapolation methods in environmental health risk assessments, *b*) identify realistic approaches for the practical application of low-dose extrapolation incorporating the relevant scientific evidence to the fullest extent feasible, and *c*) identify areas for future work.

## Workshop Issues

Participants identified two key issues specifically related to the application of low-dose extrapolation methods in risk assessments of environmental pollutants: *a*) definition of mode of action (MOA) and sufficiency of data to determine MOA and *b*) the implications, for dose–response model selection, of inter-individual variability and risk additivity from background disease processes and exposures for both cancer and noncancer outcomes.

### Defining and Assessing MOA

Using MOA information has become increasingly prominent in risk assessment. Recent U.S. EPA documents on human health risk assessment and selection of low-dose extrapolation approaches emphasize the use of MOA data in characterizing dose–response relationships for both cancer and noncancer outcomes (U.S. EPA 2004, 2005). In these documents, mode of action is contrasted with mechanism of action, with the latter term implying a more detailed understanding of key biological events, typically at the molecular level.

Comprehensive approaches for evaluating the evidence to support selection of any particular MOA have not yet been fully developed. To date, efforts of the International Programme on Chemical Safety (IPCS) (Boobis et al. 2006) and the International Life Sciences Institute (Meek et al. 2003) have focused on frameworks to evaluate MOAs and the human relevance of animal tumor data for human exposures to carcinogens. An IPCS framework for evaluating the human relevance of noncancer MOAs is under development (IPCS 2006). Well-established systematic evidence gathering, assessment, and synthesis processes have been widely applied in other domains such as clinical medicine and public health (e.g., the Cochrane Collaboration reviews, http://www.cochrane.org). Processes for the conduct of such systematic reviews of evidence are important for characterizing the strength of evidence to support an association or effect. Standard terminology developed to describe the strength of epidemiologic evidence supporting disease causation (e.g., Hill 1965) has been implemented in reports such as the U.S. Surgeon General’s reports on the health consequences of smoking (e.g., U.S. DHHS 2004). These efforts can be useful in developing a comprehensive strength-of-evidence approach for MOA both for cancer (U.S. EPA 2005) and noncancer (U.S. EPA 2008a, 2008b). Guyton et al. (2008) further elaborate on MOA definitional and evidentiary issues.

### Approaches to Low-Dose–Response Extrapolation

Participants proposed several modifications to current practice of low-dose extrapolation model selection during the workshop discussion that they generally thought better reflected recent methods in epidemiology and statistics and, in several instances, more probabilistic descriptions of risk. The first approach illustrates how MOA categories can be better conceptualized to facilitate quantitative assessment. The next three approaches are inference based (as opposed to MOA based) and include a conceptual model that explicitly incorporates both variability and uncertainty to better estimate population dose–response, arguments for linear low-dose extrapolation as the default, and inclusion of severity in the standard uncertainty factor (UF) approach. These approaches are presented approximately in decreasing order of complexity. Lastly, we discuss model averaging to better reflect uncertainty in model selection.

#### Categorical approach to low-dose extrapolation using MOA information

Advances in basic sciences are increasing biological understanding of disease processes and providing the foundation for more biologically informed frameworks of quantitative risk estimation. However, current understanding of specific biological responses from exposure to particular chemicals, and how sensitivity changes with age, is relatively limited. Understanding mechanisms of action is generally so data intensive that progress has been difficult, whereas MOAs, as currently conceptualized and described, are often so general as to not inform quantitative models.

The first conceptual approach focuses on a small number of broadly defined MOA categories, using evidence from both the chemical and general literatures on the MOA biology to develop generic dose–response models for each category. MOA categories and models could be developed by examining *a*) the reversibility of the chemical’s action on the biological system at low doses or at preclinical stages of developing biological responses and, *b*) if reversible, the rate of repair or, *c*) if irreversible, the numbers of unreversed/unrepaired damage steps needed to produce clinically detectable harm. Data gaps associated with the MOA(s) for an individual chemical could be bridged with information from other chemicals within the MOA category. For example, the approach might include three MOA categories, such as low-dose reversible mechanisms (e.g., irritation), small numbers of generally irreversible events (e.g., mutations), and large numbers of chronic cumulative, irreversible events (e.g., neuronal loss leading to Parkinsonism). It would not exclude other descriptions of MOAs, but would focus efforts on the most common and well-understood disease processes and the attributes of importance to modeling dose–response (for further discussion, see Hattis et al. 2008).

#### Inference-based models for low-dose extrapolation

The complex molecular and cellular events that underlie the actions of agents that lead to cancer and noncancer outcomes are likely to be both linear and nonlinear. At the human population level, however, biological and statistical attributes tend to smooth and linearize the dose–response relationship, obscuring thresholds that might exist for individuals. Most notable of these attributes are population variability, additivity to preexisting disease or disease processes, and background exposure–induced disease processes. (Measurement error also undoubtedly contributes to this phenomenon.) The linear appearance of the population-level dose–response function does not presume that the dose–response relationship is necessarily linear for individuals (Lutz 1990, 2001; Lutz et al. 2005), but may reflect a distribution of individual thresholds. These attributes are likely to explain, at least in part, why exposure–response models of the relationship between cancer or noncancer health effects and exposure to environmental toxicants with relatively robust human health effects databases at ambient concentrations (e.g., ozone and particulate matter air pollution, lead, secondhand tobacco smoke, radiation) do not exhibit evident thresholds, even though the MOAs include nonlinear processes for key events (NRC 2005; U.S. EPA 2006a, 2006b, 2006c; U.S. DHHS 2004). These attributes of human population dose–response relationships have been extensively discussed in the broader epidemiologic literature (e.g., Rothman and Greenland 2008) but not often in conjunction with using animal data to estimate human risks. Discussions at the workshop led to several proposed dose–response models described below, based on the above considerations.

##### Low-dose modeling incorporating uncertainty and variability

Participants discussed a quantitative modeling approach that more explicitly accounts for both uncertainty and variability in estimating low-dose risk (see Appendix 2 for expanded description). This approach can be used for cases where single or multiple MOAs are involved and a stochastic linear process dominates the biological disease process for relatively low doses. It can also be applied in cases where the slope of the dose–response relationship in the observable range may differ from the low-dose slope if, for example, multiple MOAs contributed to the dose–response relationship in the observable range but at low doses one MOA was considerably more dominant. A variant of the approach can be used to characterize population dose–response relationships resulting from the inclusion of individuals with distinct thresholds (see Appendix 2, Equations 1 and 2). This approach incorporates variables to account for uncertainties related to animal-to-human and high- to low-dose extrapolations, as well as interindividual human variation. The goal is to support statements such as, “The risk of effect does not exceed *x* level for the *y*th percentile individual, stated with confidence of *z*%.” Such expressions of risk are more robust than what is typically available.

##### Linear low-dose extrapolation from the range of observed responses

Issues regarding current default conventions typically applied to exposure–response relationships for most “toxic” pollutants (i.e., linear, no-threshold assumption for cancer outcomes and a threshold for noncancer outcomes) versus the convention used for several ambient air pollutants, secondhand tobacco smoke, and radiation (i.e., linear, no-threshold assumption for cancer and noncancer outcomes) received considerable discussion. Participants generally concurred that modeling approaches using a linear, no-threshold assumption improved consideration of the population-level factors (noted above) for both cancer and noncancer end points.

Participants noted the use of specific biological knowledge to adjust the slope of the dose–response estimate as a variant of this approach worthy of further exploration. For example, if the dose–response function was based on rodent data, and if humans were less sensitive than rodents at some key event in the disease process, then the slope of the function could be adjusted accordingly, based on comparative species sensitivities. This adjustment could be done semiqualitatively absent robust data to make precise adjustments.

##### Modification of the EPA uncertainty factor (UF) approach

The current default approach to low-dose extrapolation for noncancer outcomes divides either a no-observed-adverse-effect level (NOAEL) or a lowest-observed-adverse-effect level (LOAEL) or, preferably, a statistically calculated point of departure (an estimate of the lower end of the range of observed responses) by selected UFs, for example, a UF of 10 for human-to-animal extrapolation. A proposed modification of this approach discussed by participants includes an additional UF to account for severity of health effects, a modification that is not considered in the current approach. The primary advantage of the UF approach versus modeling is that it more effectively communicates that quantitative low-dose extrapolation is uncertain under almost any circumstance.

#### Low-dose extrapolation model selection

Model selection is one significant source of uncertainty in extrapolating low-dose risks. It has long been recognized that alternative models may lead to strikingly different estimates that vary, for example, by the assumption of a threshold or of linearity or nonlinearity (e.g., Meier et al. 1993; Portier 2000). Workshop participants discussed the concept of model averaging as a statistical approach to inform model selection. Model averaging does not require selecting a single model. It acknowledges the uncertainties associated with model selection and allows for incorporation of any prior information that would lead to particular weights for a suitable, usually large, specified set of models that are often weighted equally *a priori*. Posterior model probabilities are then computed for the individual models, reflecting the likelihood that a model holds, given the observed data. Model results are then averaged with respect to the posterior probabilities, giving greater weight to those best fitting the data (e.g., Raftery et al. 1997). External information may also be used in specifying prior weights.

## Workshop Findings

Concluding discussions provided a general consensus on answers to several key questions and highlighted areas where opportunities exist for enhancing low-dose risk extrapolation methods. These included improvements to the risk extrapolation process by refining existing, or developing new, procedures, in part drawing on approaches already used in public health decision making. Finally, participants identified gaps in the science that might be filled with a targeted research initiative.

### Harmonization of cancer and noncancer risk models and an inference-based approach

Workshop participants were uniformly of the view that the dichotomy between low-dose–response extrapolation methods typically applied to cancer and noncancer outcomes is not useful. Currently, in the absence of convincing chemical-specific evidence, low-dose linearity without a threshold is assumed for carcinogens, whereas noncarcinogens are generally considered to have thresholds. The approaches used to characterize their dose–response relationships reflect these assumptions. Participants proposed that the dichotomy be set aside and concluded that selection of low-dose extrapolation models be informed by categorization of mechanisms of toxicity, such as genotoxic, epigenetic, or cytotoxic processes, and by population-level factors (e.g., susceptibility).

Almost all workshop participants preferred a linear, no-threshold approach to low-dose extrapolation, combined with modeled estimates of the low range of the observed data (e.g., benchmark dose modeling), for both cancer and noncancer outcomes. We discuss this in more detail below. A small minority of participants expressed some reservation regarding selection of a linear nonthreshold dose–response function as the default model assumption for cancer and noncancer outcomes given information on human biologic processes such as reversibility and repair.

### MOA-based models

Although emphasis on using MOA-based models qualitatively has increased, much less attention has been given to translating the understanding of MOA into quantitative estimates or useful models for low-dose extrapolation. In the cancer paradigm, the multistage theory of carcinogenesis has provided the basis for developing corresponding statistical models. For noncancer outcomes, a broad range of specific and nonspecific MOAs are relevant to model development. Additionally, noncancer end points are likely to be complex and involve multiple interdependent physiologic changes. Interdisciplinary consideration of this issue is needed, perhaps for each of the major classes of MOAs, because many biologic processes are relevant to both cancer and noncancer health outcomes.

Using MOA to inform dose–response modeling holds substantial promise, and there are potentially numerous ways to conceptualize MOAs. Extensive multidisciplinary collaboration among biologists, toxicologists, epidemiologists, and statisticians will be needed to identify MOAs that describe the biological dose–response process in a manner that is informative for modeling. Participants recognized that successful development of this approach will likely extend beyond the near term. At least one workshop participant expressed concern that practical biologically informed dose–response modeling is unlikely for the foreseeable future owing to the complexities of disease processes.

### Inference-based model selection

Most, but not all, workshop participants concluded that for population-level risk analyses, in the absence of MOA-based dose–response models, the most appropriate low-dose extrapolation approach for both cancer and noncancer end points is linear, no-threshold extrapolation from the range of observed responses, recognizing the effects of population variability as well as additivity to background disease and exposures on the dose–response function. The inference that the population response at low doses would increase linearly with dose is drawn primarily from the impact on modeled dose–response relationships of these factors and is not inconsistent with the existence of individual thresholds. Extensive observational data to evaluate low-dose response in humans are available currently for only a few environmental agents. However, available data support use of linear, no-threshold low-dose extrapolation, unless there are sufficient data to select an alternative model.

### Assumptions regarding excess and background risks

The choice of the underlying model to use in considering how additional risk from an exposure combines with other factors (i.e., the determinants of the “background risk”) often remains uncertain. Sufficient confidence in the understanding of MOA for both the background determinants and the agent of interest is needed to address this issue with any certainty. Absent such knowledge, risk assessors often default to a multiplicative effect of the exposure of interest on background risk, in part for computational convenience. Workshop participants suggested that sensitivity analyses may be used to explore the consequences of assuming additivity to background risk or other alternatives.

Participants noted that, because of the paucity of mechanistic information to inform selection of disease categories, many mechanistically dissimilar diseases may be added incorrectly to background risks. In some situations, the additivity assumption may not be useful, for example, when toxicity is associated with high doses of essential elements or when adaptive capabilities at various levels of biological organization are sufficient to modulate responses to environmental stressors at the population level.

### Integrated models drawing on human and animal data

The workshop participants agreed that risk models might be enhanced if both human and animal data could be used collectively, to inform the model. The development of models that integrate information from both human and animal data hold potential for improving the precision of low-dose extrapolation models.

### Population variability

The theme of incorporating population variability into dose–response assessment and low-dose extrapolation was prominent in workshop discussions. The increasing understanding of the significance of interindividual variability for population-level low-dose extrapolation modeling was reflected in several of the approaches presented at the workshop.

## Workshop Recommendations

### Development of an operational definition of an MOA

Although the concept of MOA is inherently useful for risk assessment, current definitions lack specificity for selecting low-dose extrapolation models. Blurred definitional lines exist between mode of action, posed as a pragmatically useful level of understanding of “key events” involved in specific health outcomes, and mechanism of action, which implies a deeper, more deterministic knowledge of the exposure–disease process. If the MOA concept is to be universally applicable in risk assessment, the general guidance currently offered to define MOA should be made more specific and include expanded definitional language to improve application in risk assessments.

### Development of approaches for evaluating and synthesizing evidence on MOA

Workshop participants recommended extending methodology to determine the level of evidence to support a particular MOA. A standardized process that is transparent and replicable for gathering, reviewing, and synthesizing evidence should be developed. A hierarchical classification of the evidence for a particular MOA should also be developed, potentially drawing on existing approaches. This might be tested in side-by-side comparisons with several agents that have been assessed using existing approaches.

### Incorporation of variability, background incidence, and background exposures into current models

Current dose–response models could be refined to include more explicit consideration of population variability, background disease incidence, and background exposures. Population and/or chemical specific data should be used when available.

### Use of model-averaging approaches

Participants recommended Bayesian model averaging as one approach to addressing model selection and suggested model averaging as an approach for considering the consequences of analytic model selection for risk characterization.

## Areas of Suggested Further Research

### Develop and test process for characterizing level of evidence for MOA

There is a critical need to develop criteria to evaluate, and a framework to classify, strength of evidence related to determining an MOA given the increasing emphasis on using MOA-based dose–response approaches. Application of evidence criteria and the related classification framework to real-world examples of chemical case studies is necessary to ensure the approaches’ utility.

### Explore variability in the human population relative to animal populations

A better understanding of the nature of, and biologic bases for, the variability in human health responses associated with environmental contaminant exposures that can reflect differences in underlying genetic composition in populations, social factors, and/or preexisting disease status is needed to inform future improvements to dose–response models. Improved animal models of human disease states, and of genetic and physiologic variability, will also be needed to provide dose–response data relevant to the biologic variability of human populations.

### Support a methodologic research agenda

#### Apply MOA information to models

The anticipated scientific advances in understanding MOAs for classes of chemicals will require translation into dose–response models for chemical risk assessments. If realized, these scientific advances hold promise for developing relevant MOA-based categorical approaches to dose–response modeling.

#### Explore statistical approaches to model selection

Improvements to statistical approaches for model selection, such as model averaging, should be pursued. Case study applications of these advanced statistical approaches will identify potential strengths and weaknesses of these approaches and their significance for risk characterization.

#### Consider background risk

Expanded understanding of MOAs for environmental contaminants and biologic bases for disease processes could inform efforts to improve dose–response models that integrate background disease risks with pollutant exposures.

#### Develop hybrid modeling approaches

Increased efforts are needed to develop modeling approaches that integrate data for multiple species and health end points.

## Conclusions

This workshop report presents recommendations for advancing the science and practice of low-dose–response extrapolation for assessing and characterizing population health risks from exposures to environmental contaminants.

A common assumption when extrapolating experimental animal data to estimated human responses at low doses is that if nonlinear or dose-transitional key events in an MOA are identified, then an actual or practical threshold exists. Although this may be true for individuals, its applicability to large population is less certain. Population variability, as well as the potential for additivity with other preexisting background disease processes or exposures, is an inherent component of large-population dose–response relationships that influences consideration of no-threshold low-dose–response models. Therefore, it is difficult to draw conclusions about the shape of the dose–response function for the general population from MOA information alone. Well-researched examples from the epidemiologic literature regarding, for example, particulate matter, ozone, lead, secondhand tobacco smoke, and radon reinforce the need for updating dose–response assessment procedures for extrapolating dose–response models to low-dose exposures, particularly regarding continued application of the threshold dose–response as an inference-based model for noncancer outcomes.

## Appendix 1: State-of-the-Science Workshop: Issues and Approaches in Low Dose–Response Extrapolation for Environmental Health Risk Assessment, 23–24 April 2007, Baltimore, Maryland, USA

### Co-Chairs

Ila Cote, U.S. EPA, Research Triangle Park, NC; and Jonathan M. Samet, Johns Hopkins Bloomberg School of Public Health, Baltimore, MD.

### Participants

Linda S. Birnbaum, U.S. EPA, Research Triangle Park, NC; Thomas A. Burke, Johns Hopkins Bloomberg School of Public Health, Baltimore, MD; Kenny S. Crump, Environ Corp, Ruston, LA; Francesca Dominici, Johns Hopkins Bloomberg School of Public Health, Baltimore, MD; Elaine M. Faustman, University of Washington School of Public Health and Community Medicine, Seattle, WA; Mary Fox, Johns Hopkins Bloomberg School of Public Health, Baltimore, MD; Seymour Garte, University of Pittsburgh Cancer Institute, Pittsburgh, PA; Dale B. Hattis, Clark University, Worcester, MA; Ralph L. Kodell, University of Arkansas for Medical Sciences, Little Rock, AR; Frederick J. Miller, consultant, Cary, NC; Peter Preuss, U.S. EPA, Washington, DC; Louise M. Ryan, Harvard School of Public Health, Cambridge, MA; Paul D. White, U.S. EPA, Washington, DC; Ronald H. White, Johns Hopkins Bloomberg School of Public Health, Baltimore, MD; Lauren Zeise, California EPA, Office of Environmental Health Hazard Assessment, Sacramento, CA.

## Appendix 2: Linear Low-Dose Framework Incorporating Uncertainty and Variability

This model estimates low-dose human risk building on the slope of the dose–response function at the benchmark dose (Equation 1). The slope of the dose–response relationship in the observable range could differ from the low-dose slope if, for example, multiple MOAs contributed to the dose–response relationship in the observable range but at low doses one MOA was considerably more dominant. To account for this difference in low-dose slope, the slope at the benchmark dose could be modified by a factor *M**_S_*, an adjustment factor based on mechanistic understanding. When linearity is expected to dominate from the lower end of the observed dose–response relationship to lower doses, *M**_S_* takes on a value of 1. Low-dose risk could be expressed as

where Risk*_H_* is the low-dose human risk, Slope_BMD_ is the slope of the dose–response curve at what would be chosen as the benchmark dose under the current practice, *M**_S_* adjusts for the differences in slope at the high doses compared with low doses (0 ≤ *M**_S_* ≤ 1), *F**_H_*_–_*_A_* adjusts for interspecies differences, and *D* is the dose.

*F**_H_*_–_*_A_* is typically expressed as two factors to account for human–animal differences: one in pharmacokinetics and the other in pharmacodynamics: *F**_H_*_–_*_A_* = *F**_H_*_–_*_A_*_PK_ × *F**_H_*_–_*_A_*_PD_. In cases where cross-species differences in pharmacokinetics were used to derive the Slope_BMD_, the *F**_H_*_–_*_A_* would be represented by *F**_H_*_–_*_A_*_PD_.

Each of the factors in Equation 1 may represent a model, a single number, or a distribution, depending on the nature of the data and the goal of the analysis.

Persons can differ considerably in susceptibility to effects from toxicants for many reasons, including genetics, lifestyle, health status, and background exposures. Uncertainty lies in each of the factors given in Equation 1 above, as well as in our understanding of the degree of variability in susceptibility. The following provides a framework to account for both uncertainty and variability in estimating low-dose risk for Equation 1. The goal is to enable the expressions such as, “The risk of effect does not exceed *x* level for the *y*th percentile individual, stated with confidence of *z*%.”

### Uncertainty

To address the uncertainty, a random variable *U* is introduced into Equation 1 as a means of formally accounting for uncertainty:

The distribution of *U* would be specified and would depend on the analysis being performed. An alternative, and the conventional, approach would be to simply express each factor in Equation 1 as a random variable, and not introduce *U*.

In certain cases, it may be convenient and appropriate to describe uncertainty in Risk*_H_* mathematically with a lognormal distribution as a default if, for example, the uncertainty in each of the factors in Equation 1 can be represented by a lognormal distribution. In this case, Equation 2 may be restated as

where the random variable *u* is distributed normally with mean 0 and variance σ^2^. Thus, for this simplistic case,

### Variability

For some toxicity processes with multiple MOAs, such as cancer, which involve cell proliferation and mutation MOAs, the low-dose linearity may be dominated by a stochastic process that results in linear dose–response relationships for exposed individual persons. In this case, variability and *M**_S_* may be essentially independent at low doses. In this case, the risk for the *y*th percentile individual is given by

where *V**_H y_*_th_ is the *y*th quantile of the distribution that describes the ratio of the *y*th percentile individual to the median individual. If the uncertainty in the *V**_H y_*_th_ and the other elements of the uncertainty are described by a lognormal distribution, the overall uncertainty represented by σ^2^ would be described by adding a term σ_log*V*^2^_ to the terms given in Equation 4 above. This would not necessarily be the case if the low-dose linearity arises at the population level from processes that at the individual level involve threshold responses. The *M**_S_* and variability *V**_H_* would then not be independent. In this case, descriptions of population risk and uncertainty using Equations 1 and 2 could be developed, as well as exploration of risk in the more susceptible segments of the population.
